# Evolutionary genomic remodelling of the human 4q subtelomere (4q35.2)

**DOI:** 10.1186/1471-2148-7-39

**Published:** 2007-03-14

**Authors:** Beatrice Bodega, Maria Francesca Cardone, Stefan Müller, Michaela Neusser, Francesca Orzan, Elena Rossi, Elena Battaglioli, Anna Marozzi, Paola Riva, Mariano Rocchi, Raffaella Meneveri, Enrico Ginelli

**Affiliations:** 1 Department of Biology and Genetics for Medical Sciences, University of Milan, Milan, Italy; 2 Department of Genetics and Microbiology, University of Bari, Bari, Italy; 3 Biology II – Anthropology and Human Genetics, University of Ludwig Maximilians, Munich, Germany; 4 Department of Experimental Medicine, University of Milan-Bicocca, Monza, Italy

## Abstract

**Background:**

In order to obtain insights into the functionality of the human 4q35.2 domain harbouring the facioscapulohumeral muscular dystrophy (FSHD) locus, we investigated in African apes genomic and chromatin organisations, and the nuclear topology of orthologous regions.

**Results:**

A basic block consisting of short D4Z4 arrays (10–15 repeats), 4q35.2 specific sequences, and approximately 35 kb of interspersed repeats from different LINE subfamilies was repeated at least twice in the gorilla 4qter. This genomic organisation has undergone evolutionary remodelling, leading to the single representation of both the D4Z4 array and LINE block in chimpanzee, and the loss of the LINE block in humans. The genomic remodelling has had an impact on 4qter chromatin organisation, but not its interphase nuclear topology. In comparison with humans, African apes show very low or undetectable levels of *FRG1 *and *FRG2 *histone 4 acetylation and gene transcription, although histone deacetylase inhibition restores gene transcription to levels comparable with those of human cells, thus indicating that the 4qter region is capable of acquiring a more open chromatin structure. Conversely, as in humans, the 4qter region in African apes has a very peripheral nuclear localisation.

**Conclusion:**

The 4q subtelomere has undergone substantial genomic changes during evolution that have had an impact on chromatin condensation and the region's transcriptional regulation. Consequently, the 4qter genes in African apes and humans seem to be subjected to a different strategy of regulation in which LINE and D4Z4 sequences may play a pivotal role. However, the effect of peripheral nuclear anchoring of 4qter on these regulation mechanisms is still unclear. The observed differences in the regulation of 4qter gene expression between African apes and humans suggest that the human 4q35.2 locus has acquired a novel functional relevance.

## Background

The distal portion of the human 4q35 genomic region (4q35.2) contains a complex arrangement of repetitive sequences and several genes including facioscapulohumeral muscular dystrophy (FSHD) region genes 1 and 2 (*FRG1 *and *FRG2*) [1]. A polymorphic tandem array of 3.3 kb repeats (D4Z4) has also been detected distally to these genes [2]; the D4Z4 unit contains an open reading frame (ORF) encoding a homeobox protein (DUX4), but DUX4 transcription has not been demonstrated [3,4].

Deletions within the subtelomeric D4Z4 array lead to autosomal-dominant facioscapulohumeral muscular dystrophy (FSHD MIM 158900) [2,5]. Unaffected individuals have 11–100 copies of the D4Z4 repeat on both chromosomes 4q, whereas almost all FSHD patients have one chromosome 4q characterised by 1–10 tandem copies [3]. Moreover, 4q35.2 D4Z4 repeats are methylated in the general population, whereas the contracted array is hypomethylated in FSHD patients [6,7]. The property of 4qter array methylation has led to the hypothesis that the pathogenesis of FSHD is associated with an epigenetic mechanism. In this regard, the array contraction might cause proximal or distal changes in chromatin structure, with the consequent up-regulation of one or more 4qter genes [8], and it is known that transgenic mice over-expressing the *FRG1 *gene (located 125 kb from the D4Z4 array) develop a muscular dystrophy resembling human FSHD.

Some studies of inappropriate gene activation in the FSHD region support this hypothesis [9-11], but other papers report a similar level of 4qter gene expression in FSHD patients and controls [12,13]. Furthermore, recent findings indicate that FSHD may share some features with nuclear envelope dystrophies [14-16]. Unlike most other chromosome ends, normal and FSHD 4qter alleles are preferentially localised at the nuclear rim [14,15]. It is assumed that this consistent and non-random localisation of 4qter in the periphery of human cell nuclei has some functional significance, and that FSHD may be due to improper interactions with transcription factors or chromatin modifiers at the nuclear envelope [15].

Despite these observations, studies aimed at defining the molecular mechanisms underlying the pathogenesis of FSHD are difficult to perform because the genomic sequences involved in the disease are a complex patchwork of duplications. The D4Z4 repeat is not restricted to chromosome 4; perfect arrays of D4Z4 units can also be detected on chromosome 10q, and there are additional homologous sequences interspersed with beta satellites on many heterochromatic loci, such as the short arms of acrocentric chromosomes and the pericentromeric region of chromosome 1q [17]. Moreover, a subset of 4q35.2 sequences proximal and distal to the D4Z4 array, including the *FRG1 *and *FRG2 *genes, are duplicated in the human genome [4,18,19]. This complex genomic scenario makes it difficult to understand the regulation of gene expression at 4q35.2, as well as its alteration in FSHD.

In order to obtain further insights into the function of the human 4qter genomic region, we studied its evolution by investigating genomic organisation, nuclear positioning, chromatin acetylation levels and gene expression in African apes. We chose the gorilla as a starting point because previous studies have indicated the presence of a chromosome-specific block of subtelomeric sequences in 4qter [20], and D4Z4 dispersion is less complex than in humans.

## Results

### Isolation and sequence characterisation of gorilla 4qter genomic clones

A gorilla genomic library was screened using a probe from a human subtelomeric block of sequences (defined as block 3 in [20]) that are located on chromosomes 1p, 8q, 15q and 19p in the human genome, but only on chromosome 4qter in gorilla and chimpanzee. A non-repetitive hybridisation probe was generated by PCR on DNA from BAC AC140725 mapped to 15q26.3, which includes subtelomeric block 3 [20] (Additional file 1). By this approach, six BAC clones were obtained. In the human reference sequence, one or both ends of five clones (CH255-11C6, CH255-18C5, CH255-23B19, CH255-39M12, and CH255-41H7) were similar within a region of approximately 35 kb on chromosomes 15q26.3 and 19p13.3 [21] (Additional file 1 and Additional file 2), which proved to be a complex patchwork of fragments from different LINE subfamilies (the LINE block) (Additional file 3). Subtelomeric block 3 and the LINE block in the human reference sequence overlap for ~10 kb (Additional file 1). Furthermore, one end of three clones (CH255-23B19, CH255-39M12 and CH255-39N14) showed similarity with the 4q35.2 locus, within a region of approximately 15 kb distal to the *FRG2 *gene (Additional file 2).

After KpnI or EcoRI digestion and hybridisation with D4Z4 (LSau probe), all of the clones showed a very similar restriction and hybridisation pattern of bands (Additional file 4). These results strongly suggested that all of the isolated BACs carry very similar DNA sequences, including an array of approximately 10–15 D4Z4 repetition units, as derived from the densitometric profile of KpnI-digested BAC DNAs.

The sequence content of the gorilla BAC clones was investigated by means of orthologous PCR using primer pairs derived from the human reference sequence that defined the *FRG1 *and *FRG2 *promoters and marker 13E11 at 4q35.2, and 35 kb of the LINE block at 15q26.3 (the primer pairs are listed in Additional file 5). All of the clones were remarkably similar insofar as they included the *FRG2 *promoter, the 13E11 marker, and an almost complete LINE block of 35 kb (Additional file 6). The isolated gorilla genomic clones thus showed redundancy of essentially three classes of sequences: an array of D4Z4 repeats, a LINE block, and a region of non-repetitive 4q35.2 DNA between the *FRG2 *gene and the 13E11 marker (approx. 40 kb). Taken together, the molecular analyses strongly suggested that the isolated genomic sectors genuinely derived from the gorilla 4q35.2 locus.

### Chromosomal evolution of the 4qter region

**Figure 1:**
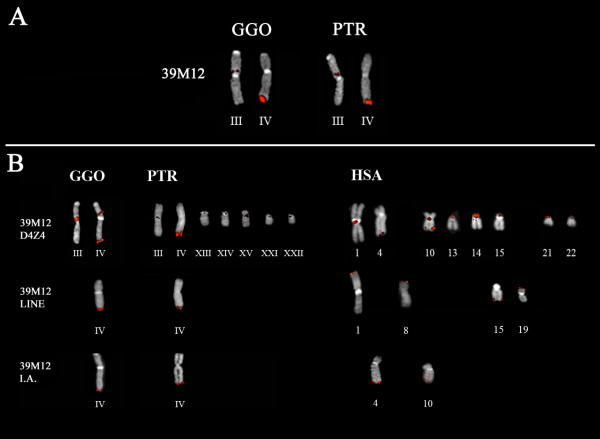
Chromosomal location of gorilla sequences in the BAC **CH255**-39M12 in different primate species revealed by means of comparative FISH experiments on gorilla (GGO), chimpanzee (PTR) and human (HSA) metaphase chromosome spreads. (**A**) FISH hybridisation of BAC **CH255**-39M12 on GGO and PTR metaphases; (**B**) FISH on GGO, PTR and HSA chromosomes with gorilla D4Z4 unit (39M12-D4Z4), LINE (39M12-LINE) and **CH255**-39M12 Inter-Alu (I.A.). The Roman numbers refer to the homologous human chromosome.

Four BAC clones (CH255-18C5, CH255-23B19, CH255-39M12, and CH255-39N14) were mapped by FISH on gorilla metaphase chromosome spreads, and essentially detected 4qter and an interstitial location on chromosome 3p (Fig. 1 and Additional file 7); comparable results were obtained on chimpanzee chromosome spreads (Fig. 1 and Additional file 7). Furthermore, inter-Alu sequences from BACs CH255-18C5 and CH255-39M12, yielded a single FISH signal at 4qter on African ape chromosomes (Fig. 1 and Additional file 7), whereas both probes hybridized to 4qter and 10qter on human chromosomes (Fig. 1 and Additional file 7).

The chromosomal distribution of D4Z4 and the LINE block was derived using probes consisting of a 3.3 kb unit (39M12-D4Z4) and a LINE fragment of ~10 kb (39M12-LINE) obtained from BAC CH255-39M12. The LINE probe hybridised only the gorilla and chimpanzee 4qter region, whereas four subtelomeric regions were highlighted on human chromosomes (1p, 8p, 15q and 19p) (Fig. 1 and Additional file 7). Conversely, the D4Z4 repeats showed a more pronounced spread from gorilla to humans (Fig. 1 and Additional file 7).

### Genomic organisation of the gorilla 4qter region

**Figure 2:**
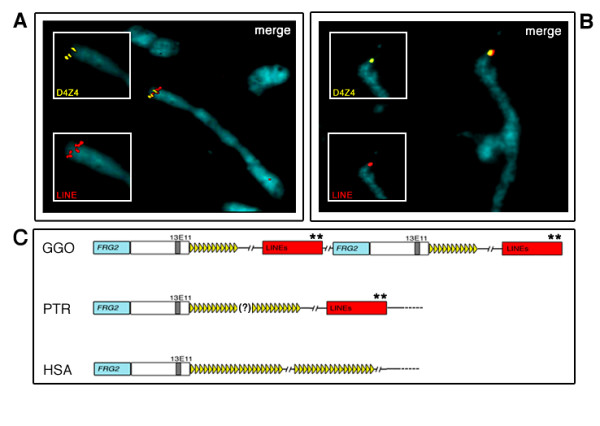
Localisation of D4Z4 and LINE sequences on gorilla (GGO) (**A**) and chimpanzee (PTR) (**B**) 4q stretched chromosomes. The reciprocal localisation was derived by dual colour FISH using a gorilla D4Z4 unit (yellow signals) and a LINE probe (red signals). Figures A and B show the hybridisation patterns obtained using a single probe (D4Z4 and LINE inserts), and the merged hybridisation (merged). **C) **Schematic representation of gorilla (GGO), chimpanzee (PTR) and human (HSA) 4qter genomic organisations. The gorilla and chimpanzee organisations were derived from the molecular and FISH data herein, whereas the human organisation is taken from previous studies (for a review, see van der Maarel *et al*., 2006). The question mark (?) indicates that the length of the D4Z4 array in chimpanzee was not determined. Double asterisks (**) indicate the overlapping region between the LINE block and subtelomeric block 3 in the human genome [20].

As the six gorilla BAC clones were isolated from one genome equivalent and showed a very similar internal sequence organisation, we speculated that the gorilla 4qter region might consist of tandem repetitions of a stretch of DNA made up of 4q35.2-specific sequences linked to a short array of D4Z4 and spaced by a block of LINE sequences. To verify this hypothesis, we carried out two-colour FISH experiments using 39M12-D4Z4 and 39M12-LINE probes on stretched gorilla metaphase chromosomes. As shown in Figure 2A, both probes yielded multiple hybridisation spots at the 4q subtelomere, which were clearly intermingled, and similar results were also obtained using BAC 39M12 as a probe (data not shown); conversely, the D4Z4 and LINE probes gave single hybridisation signals on stretched chimpanzee chromosomes (Fig. 2B). Figure 2C shows the proposed organisation of the 4qter region of human, chimpanzee and gorilla based on these results.

### Nuclear topology of the 4qter region in African apes

**Figure 3:**
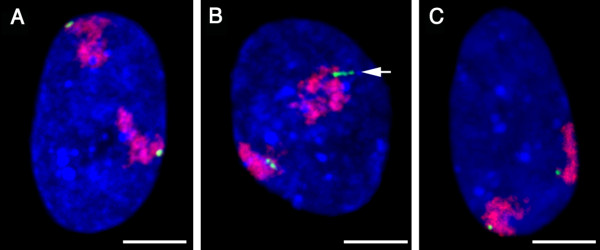
Maximum intensity z-projections of representative gorilla and chimpanzee fibroblast nuclei after 3D-FISH with BAC 39M12 (green) and a chromosome 4 painting probe (red). The chromosomal counterstain is shown in blue (scale bar = 5 μm). **A) **and **B) **gorilla nuclei (A with both alleles located at the nuclear edge, and B with one allele showing a "track" of hybridisation signals – arrow); **C) **chimpanzee nucleus with one 4q35.2 allele abutted against the nuclear envelope.

To study the impact of evolutionary genomic changes in the 4qter region on nuclear topology, we performed FISH experiment using 3D-preserved fibroblast nuclei of chimpanzee and gorilla. The FISH probes were gorilla BAC clone 39M12 for the 4q subtelomere and a human chromosome 4 painting probe; in gorilla and chimpanzee interphase nuclei, we observed two major hybridisation signals per nucleus with BAC 39M12 (Fig. 3).

Visual inspection of 3D image stacks from 29 gorilla nuclei revealed 36% of 4qter signal loci abutted to the nuclear envelope defined by the border of nuclear counterstaining (Fig. 3A). One allele in 7/29 cells (~24%), and both alleles in a further seven cells (~24%), were found at the nuclear edge (Fig. 3A). In addition, 10/29 nuclei showed multiple localised hybridisation signals for one allele per nucleus, which were clearly distinguishable from the double-dot BAC signals in replicated loci (Fig. 3B). Visual inspection of confocal image stacks from 28 chimpanzee nuclei identified one allele at the nuclear edge in 11/28 cells (~39%) and both alleles at the nuclear edge in 4/28 cells (~14%): a total of 34% of alleles (Fig. 3C). All of the BAC signals were dot-shaped; there were no multiple localised hybridization signals like those seen in the gorilla nuclei.

**Figure 4:**
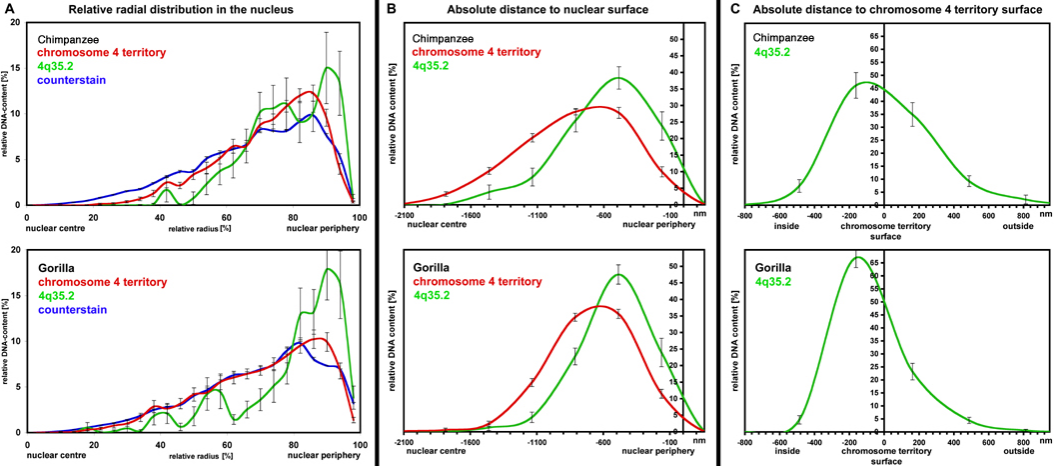
Quantitative evaluation of the radial distributions of chromosome 4 territories and 4q35.2 loci in 28 chimpanzee and 29 gorilla fibroblast interphase nuclei. **A) **Relative radial distribution (0% = nuclear centre; 100% = nuclear envelope), **B) **Absolute distance to nuclear surface, and **C) **Absolute distance to chromosome 4 territory surface.

Quantitative evaluation of the radial arrangement by means of 3DRRD and ADS software confirmed the clearly peripheral nuclear location of the gorilla locus syntenic to human chromosome 4q35.2 (Fig. 4). The average relative radius (ARR) for 29 evaluated nuclei was 79.2% (0% = nuclear centre and 100% = nuclear edge), equivalent to an approximately 440 nm mean distance from the nuclear edge (Fig. 4A and 4B). Furthermore, the 4q35.2 locus was located significantly more peripherally than the chromosome 4 territory (CT) (P = 0.002 and P =< 0.001). With respect to the chromosome 4 territory surface, the BAC clone was again found very peripheral, being located on the surface, or even outside the "core" territory as defined by chromosome painting (an average 0 nm from the CT surface) (Fig. 4C).

As in gorilla, quantitative evaluation revealed the clearly peripheral nuclear localisation of the chimpanzee 4qter (Fig. 4). The ARR for 28 evaluated nuclei was 78.3%, corresponding to a mean distance of 510 nm from the nuclear edge (Fig. 4A and 4B). Furthermore, as in gorilla, the 4qter region was significantly more peripheral than the chromosome 4 territory (P = 0.020 and P = 0.001). Notably, the distribution curve obtained using 3DRRD software revealed two peaks in the nuclear periphery at approximately 75% ARR and 90% ARR, thus indicating two distinct preferential locations for the BAC signals. With respect to the chromosome 4 territory surface, the BAC clone was preferentially located at the surface or outside the "core" territory (an average of 60 nm outside) (Fig. 4C).

### Chromatin organisation and gene expression of the 4qter region in African apes and humans

**Figure 5:**
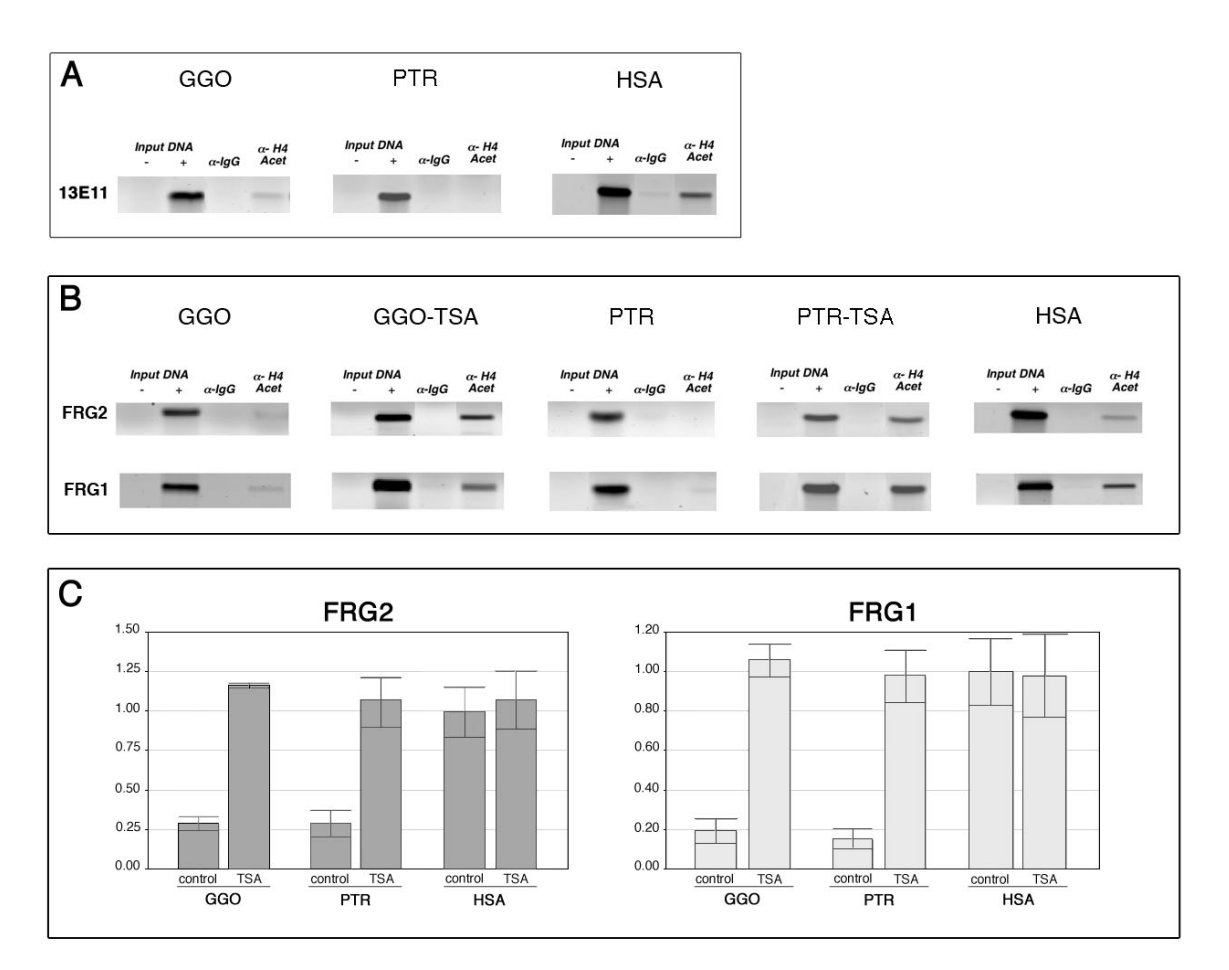
*FRG1 *and *FRG2 *histone acetylation and transcription in African apes and humans. **A) **Chromatin immunoprecipitation experiments on gorilla and chimpanzee lymphoblastoid cell lines before (GGO and PTR) and after Trichostatin A treatment (GGO-TSA and PTR-TSA). Chromatin was immunoprecipitated with a rabbit antiserum anti-acetyl histone H4, and a normal rabbit IgG as pre-immune (Upstate Biotechnology); the *FRG2 *and *FRG1 *promoter regions were amplified on Input DNA, pre-immune and H4Ac-immunoprecipitated samples using the primer pairs shown in Additional file 5. **B) **Quantification by real-time RT-PCR of the mRNA of the *FRG2 *and *FRG1 *genes; total RNA from untreated (control) and TSA-treated (TSA) GGO, PTR and HSA fibroblast cell lines was used as a template with primer pairs for *FRG1 *and *FRG2 *mRNAs. Each experiment was performed in triplicate (bars show SD).

By means of chromatin immunoprecipitation (ChIP) assays of gorilla, chimpanzee and human lymphoblastoid cell lines, we analysed the histone H4 acetylation status of the 13E11 marker proximal to the D4Z4 array, and the *FRG1 *and *FRG2 *promoters (the primer pairs are listed in Additional file 5, and their validation criteria are described in Methods and Additional file 8). The 13E11 marker and *FRG1 *and *FRG2 *promoters showed much less H4 acetylation in gorilla and chimpanzee than in human cells (Fig. 5A and 5B).

Expression analysis of the *FRG1 *and *FRG2 *genes by means of quantitative RT-PCR confirmed the results of the ChIP analysis: the *FRG1 *and *FRG2 *genes were significantly (75%) less expressed in gorillas and chimpanzees than in humans (Fig. 5C). However, treatment with the histone deacetylase inhibitor Trichostatin A (TSA) increased both histone H4 acetylation, and *FRG1 *and *FRG2 *expression, in gorilla and chimpanzee cells to levels that were comparable with those observed in human cells (Fig. 5B and 5C). This may indicate the presence of repressive complexes in the gorilla and chimpanzee 4q subtelomere that are absent from the human orthologous region, an interpretation that is supported by the analysis of *FRG1 *and *FRG2 *gene expression in TSA-treated human cells (Fig. 5C).

## Discussion

In this study, we investigated in African apes the genomic and chromatin organisation of regions syntenic to the human chromosome 4q35.2 harbouring the FSHD locus, concentrating on the D4Z4 array that has been previously suggested to be the pivotal sequence motif for human 4q35.2 transcriptional regulation [22]. As most hominoid species show considerable trans-chromosomal duplication and spreading of D4Z4 repeats, we analysed the gorilla species because it has only a few D4Z4 chromosomal locations [4].

Genomic analysis of the gorilla locus syntenic to human chromosome 4q35.2 revealed clear differences in organisation in comparison with humans. A basic block of short D4Z4 arrays (10–15 repeats) and 4q35.2-specific sequences (i.e. the *FRG2 *gene and the 13E11 marker) spaced by LINE is repeated at least twice in gorilla, whereas chimpanzee has a single D4Z4 array linked to a LINE block, and humans show further remodelling involving the loss of the LINE block. The 4q subtelomere therefore underwent substantial genomic changes during evolution, and its remodelling essentially involved D4Z4 and LINE sequences (see Fig. 2C).

In humans, the LINE block is still present at the subtelomere of chromosomes 1p, 8p, 15q and 19p, which suggests that at least the first round of duplications preceded its deletion at 4q35.2. This was a recent evolutionary event that might be mediated by the instability of subtelomeric regions, which are well known to be prone to reciprocal translocations [20,23], whereas the dispersal of the D4Z4 array from the 4q ancestral locus of the macaque has an older evolutionary history, as it took place in an ancestor of the orang-utan and preferentially involved acrocentric chromosomes in both chimpanzee and man [4,17,24]. Furthermore, 1qcen, 10qcen and 10qter are human-specific sequence dispersals of D4Z4 repeats.

Although the sequence dispersal of D4Z4 repeats during evolution preceded that of the LINE block, their simultaneous occurrence at gorilla and chimpanzee 4qter may explain the evolutionary remodelling of the region. Both sequence types are prone to unequal homologous recombinations leading to the expansion/contraction of the 4q subtelomere, and this mechanism could account for the expansion of the D4Z4 array and the complete loss of the LINE block in man.

Despite the evolutionary genomic remodelling of the 4q subtelomere, both gorilla and chimpanzee lymphoblastoid cells showed very little or undetectable histone H4 acetylation at the 13E11 marker, and the *FRG1 *and *FRG2 *gene promoters; furthermore, this was paralleled by very low levels of *FRG1 *and *FRG2 *mRNA expression. Conversely, human lymphoblastoid cells showed higher levels of both *FRG1 *and *FRG2 *promoter acetylation and mRNA expression.

The human cell data partially agreed with the results of previous experiments comparing histone H4 acetylation in lymphoblastoid and fibroblast cells from normal individuals and FSHD patients, which led to the conclusion that the observed acetylation levels across the 4q35.2 region were not like those in condensed chromatin [13]. Therefore, the low acetylation levels in gorilla and chimpanzee cells indicate the occurrence of a more condensed chromatin structure at 4qter, and this correlates well with their very low expression of *FRG1 *and *FRG2 *genes.

However, the inhibition of histone deacetylase activity in African apes restored the expression of *FRG1 *and *FRG2 *to levels comparable with those found in human cells, and this reversibility of histone acetylation makes it possible to speculate that the 4q subtelomeric region in certain gorilla and chimpanzee cell differentiation lineages could acquire a more open chromatin structure. Within the gorilla and chimpanzee locus syntenic to human chromosome 4q35.2, LINE may represent candidate sequences for chromatin condensation as the silencing of gene expression has been associated with an epigenetic mechanism mediated by long and short interspersed repeats [25]. In this regard, LINE enrichment has been correlated with the spread of X chromosome inactivation [26].

Previous studies of normal and FSHD human nuclei have shown that the 4q subtelomere is consistently localised at the nuclear periphery [14,15]. A similar analysis of 3D-preserved chimpanzee and gorilla fibroblast nuclei showed a considerable evolutionary conservation of the nuclear positioning of the chromosome 4 territory and 4q subtelomere. We found that, as in human fibroblast nuclei [14,15,27], the chromosome 4 territory in chimpanzee and gorilla is located in the nuclear periphery, and the locus syntenic to human chromosome 4q35.2 has an even more peripheral location near or at the nuclear edge. This extremely peripheral location cannot be simply explained by the current hypothesis suggesting that local gene density determines the nuclear positioning of a locus [28], because the local gene density of the 4qter region (7.6 genes/Mb in a 3 Mb window, build 36.2 [29]) is higher than that of the entire chromosome territory (6.00 genes/Mb).

In addition to the remarkable similarities in the spatial arrangement of the 4q subtelomere in humans and African apes, we also found some notable qualitative differences in the topology of chimpanzee and gorilla nuclei. In particular, the 4q subtelomere in chimpanzee nuclei showed two peripheral but distinctly different preferential positions, which may indicate the occurrence of 4q allelic variants [12].

Localization to the nuclear periphery is generally associated with gene repression [30], and the condensed chromatin structure and low expression of 4qter genes in gorilla and chimpanzee are in line with this. Conversely, although maintaining the preferential association of the 4q35.2 locus with the nuclear periphery, human cells show less chromatin condensation and higher 4q35.2 gene expression. On the basis of these data, we hypothesise that the peripheral localisation of the 4qter region is maintained independently of: a) the underlying DNA sequences (i.e. D4Z4 and LINE), b) their genomic organisation; and c) the expression of embedded genes. However, the evolutionary conservation of 4qter nuclear topology suggests that still unidentified subtelomeric DNA sequences are responsible for the preferential nuclear location, or still unknown epigenomic features of the nuclear periphery play a role in the sub-localisation of 4qter in African apes and humans.

The chromatin condensation and nuclear anchoring of the 4q subtelomere in gorilla and chimpanzee probably reflect a need to repress genes actively in a lineage-dependent manner, and sequester them in a particular nuclear sub-compartment capable of maintaining the repressive state. The LINE-mediated heterochromatisation of the African ape 4qter region could be triggered in *trans *by interaction with constitutive heterochromatin-associated proteins residing at the inner nuclear membrane [30].

In human cells, the genes of the FSHD region could be protected from repression by the loss of the LINE block in association with the expansion of the D4Z4 array and, in this regard, it has been suggested that D4Z4 may be a barrier or insulator against heterochromatin-mediated gene silencing [22,31]. Interestingly, mutations in the lamin A-type gene (*LMNA*) that cause human diseases called "laminopathies" can also abrogate the nuclear peripheral location of the 4q subtelomere [14], and so 4q35.2 gene expression might be dictated by the interaction of chromatin and/or DNA with components lining the inner nuclear membrane, such as the lamins known to recruit transcriptional regulators [32]. However, it is still not possible to rule out the possibility that repressive histone marks are responsible for the fine-tuning of human 4q35.2 gene expression.

## Conclusion

Our study showed a particular genomic organisation of the gorilla locus syntenic to human chromosome 4q35.2. During evolution, this chromosome region has undergone extensive remodelling in both chimpanzees and humans. Particularly in human cells, 4q35.2 genomic remodelling had an impact on chromatin condensation and the regulation of *FRG1 *and *FRG2 *gene expression, but did not influence the peripheral nuclear positioning of 4qter. These data suggest that evolutionary remodelling has probably conferred a novel functional relevance to the human 4q35.2 locus, and reinforce the idea that most biological phenomena can be better understood if studied in an evolutionary frame.

## Methods

### Genomic library screening

The genomic library was obtained from BAC/PAC Resources, Children's Hospital, Oakland Research Institute (CHORI-255: Gorilla BAC Library). High-density arrayed BAC filters were hybridised according to the instructions provided by BAC/PAC Resources [33] with a ^32^P-labelled non-repetitive probe obtained by means of PCR (for primers, see Additional file 5) from human BAC clone AC140725 mapping to 15q26.3 and containing subtelomeric block 3 [20] after repeat masking [34]; the filter was also hybridised with the human LSau probe pA1 [35], and the genomic clones positive to both probes were selected.

### DNA isolation and Southern blot hybridisation

The BAC clones were grown in LB medium supplemented with chloramphenicol. The DNA was purified using a Sigma PhasePrep BAC kit (Sigma Aldrich, USA), digested with restriction enzymes (Biolabs, USA), fractionated by means of agarose gel electrophoresis, blotted onto nylon filters (Hybond N+, Amersham, UK), and hybridised with a ^32^P-labelled probe representative of D4Z4 (pA1) and beta satellite sequences (A17a plasmid) [36]. Molecular hybridisation was performed in 2 × SSC at 60°C overnight, and the filters were washed at 60°C in 1 × SSC twice for 20 minutes. The hybridisation signals were quantified by means of phosphoimaging (Typhoon 9200, Amersham).

### Polymerase chain reaction, cloning and sequencing

Polymerase chain reactions were carried out using the primer pairs listed in Additional file 5, and respectively derived from the human genomic 4q35.2 region and the sequenced GGO BAC ends showing similarities with the human subtelomeric LINE block. The PCR mixtures of 25 μl included 100 ng of DNA, 0.4 μM of each primer, 0.2 mM dNTP mix, and 1 U of *Red Taq *DNA polymerase in standard reaction buffer (Sigma Aldrich, USA). The PCR conditions generally consisted of one preheating cycle at 95°C for 3 min, followed by 30 cycles of denaturation at 95°C for 30–60 s, annealing at 46°C–65°C for 30 s, and extension at 72°C for 30 s – 2 min. Inter-Alu PCR was carried out as previously described [37]. For long PCR amplification, a 50 μl reaction mixture was used according to the manufacturer's instruction (Roche, Taq Expand 20 kb), and the primers were designed on gorilla BAC end sequences (Additional file 5). Amplification was performed under the following conditions: 2 min at 92°C for initial denaturation, followed by 10 cycles of 10 s at 92°C and 20 min at 68°C, and an additional 20 cycles of 10 s at 92°C, 20 min with autoextension of 10 s per cycle at 68°C, and 7 min at 68°C for final elongation. A D4Z4 unit was sub-cloned in BSK vector from BAC CH255-39M12 after KpnI digestion and completely sequenced using the primers shown in Additional file 5 and a Big Dye terminator 3.1 system (Perkin Elmer Applied Biosystems, Norwalk, Conn) following the manufacturer's instructions. The nucleotide sequence is available at [38], with accession number: ED549314.

T7/Sp6 BAC ends were sequenced using the Big Dye terminator 3.1 system as described by BAC/PAC Resources [33]. All of the fluorescent traces were analysed using the Applied Biosystem Model 3100 DNA Sequencing System (Applied Biosystem). DNA sequence analysis was performed using DNASTAR software and NCBI facilities [38]. The newly derived nucleotide sequences are available at [38] with accession numbers: ED549315 (CH255-11C6/Sp6), ED549316 (CH255-11C6/T7), ED549317 (CH255-18C5/Sp6), ED549318 (CH255-18C5/T7), ED549319 (CH255-23B19/Sp6), ED549320 (CH255-23B19/T7), ED549321 (CH255-39M12/Sp6), ED549322 (CH255-39M12/T7), ED549323 (CH255-39N14/Sp6), ED549324 (CH255-39N14/T7), ED549325 (CH255-41H7/Sp6), ED549326 (CH255-41H7/T7).

### Fluorescent in situ hybridisation (FISH) on metaphase chromosome spreads

The metaphase chromosome spreads were obtained by standard methods from peripheral blood lymphocytes of normal human (HSA) donors, and from the primate lymphoblastoid or fibroblast cell lines *Gorilla gorilla *(GGO) and *Pan troglodytes *(PTR). The primate cell lines were provided by M. Rocchi [39]. The probes were labelled with digoxigenin-dUTP or biotin-dUTP (Roche Diagnostic) using a nick translation kit (Roche Diagnostic), and detected using fluorescein-streptavidin (SIGMA) for the biotin-labelled probes, and a CY3-anti-digoxigenin conjugated antibody (Jackson ImmunoResearch Laboratories) for the digoxigenin-labelled probes. The chromosomes were counterstained with propidium iodide and DAPI in antifade (Vectashild), and then visualised using a Leitz DM-RB microscope equipped for DAPI and FITC/TRITC epifluorescence optics. Hybridisations were performed in 50% formamide (v/v), 10% dextran sulfate, 2 × SSC at 37°C, in the presence of human Cot1 DNA (Gibco-BRL). Post-hybridisation washing included 50% formamide, 2 × SSC at 42°C, followed by three washes in 1 × SSC at 60°C (HSA), or 50% formamide, 1 × SSC at 37°C, followed by three washes in 1 × SSC at 42°C (GGO and PTR). The chromosomes were stained with DAPI (4',6-diamidino-2-phenylindole), and digital images were captured using a Leica DMRXA epifluorescence microscope equipped with a cooled CCD camera (Princeton Instruments). The fluorescence signals were recorded separately as gray-scale images. The images were pseudocoloured and merged using Adobe Photoshop software.

### Stretched chromosome preparation

The chromosomes were mechanically stretched as previously described [40]. Aliquots of 10^3^–10^6 ^mitotically active lymphoblast cells were washed in PBS and resuspended in the hypotonic solution (10 mM Hepes, 30 mM glycerol, 1.0 mM CaCl_2 _and 0.8 mM MgCl_2_) for ten minutes. Aliquots of 0.5 ml of the hypotonic cell suspensions were centrifuged (Cytospin 2, Shandom) onto clean glasses at 800 rpm for four minutes, and fixed in -20°C methanol for 30 minutes.

### FISH to 3D-preserved interphase nuclei

The gorilla (*Gorilla gorilla*) and bonobo (*Pan paniscus*) fibroblast cell lines with normal diploid karyotypes (data not shown) were kindly provided by W. Schempp, University of Freiburg, Germany. The protocol for the 3D-FISH experiments has been previously described [41]. The cells were fixed in 4% paraformaldehyde (PFA) in 1 × PBS for 10 min. The permeabilisation steps included treatment with 0.5% Triton-X100 (20 min), 20% glycerol in PBS for at least one hr, repeated freeze/thawing in liquid nitrogen, and incubation in 0.1 N HCl (5 min) and pepsin solution (2.5 mg/ml pepsin in 0.01 N HCL at 37°C for 5–10 min). The human chromosome 4 painting probe was labelled with DOP-PCR in the presence of TAMRA-dUTP, and the BAC clone 39M12 with Biotin-dUTP (Roche) by nick-translation, and 400 ng-2 μg of both probes were mixed with a 10-fold excess of human Cot-1 DNA. *In situ *hybridisation was performed for 48 hours, followed by stringency washes for 3 × 5 min in 0.1 × SSC (60°C). The biotinylated probe was detected with Avidin-Alexa488 (Molecular Probes). The 3D-fixed nuclei were counterstained for 5 min with 1 μM ToPro-3 (Molecular Probes), and visualised using a three-channel laser scanning confocal microscope (Leica SP-1; Leica Microsystems). The nuclei were scanned with an axial distance of 300 nm between consecutive light optical sections, and the confocal image stacks were processed with ImageJ software [42]. Quantitative 3D evaluation was carried out using the voxel-based software algorithms 3D-RRD (Three Dimensional Relative Radius Distribution) [43] and eADS (enhanced absolute 3D distances to surfaces) Significant differences (P = 0.05) between radial probe distributions were determined using the Mann-Whitney rank sum test (U-test).

### Chromatin immunoprecipitation (ChiP) assays

About 6 × 10^6 ^cells were treated with 1% formaldehyde and sonicated in 600 μl of protease inhibitor-containing buffer, and then the chromatin was immunoprecipitated with a rabbit anti-serum anti-acetyl histone H4 (Upstate Biotechnology) essentially according to the manufacturer's specifications. The input DNA was an aliquot of the supernatant from each centrifuged sonicate (DNA size range: about 200–600 bp), and the pre-immune chromatin was immunoprecipitated with normal rabbit IgG (Santa Cruz Biotechnology). Pre-clearing before the addition of antibody was carried out for 1 h at 4°C with constant agitation using 10 μl of tRNA (20 mg/ml), 20 μl salmon sperm DNA (10 mg/ml) and 20 μl protein G-agarose beads added to 100 μl sample for each immunoprecipitation. The immunoprecipitates were collected for 3 h at 4°C with constant agitation using 10 μl of tRNA (20 mg/ml), 20 μl salmon sperm DNA (10 mg/ml) and 20 μl protein A-agarose beads added to the 1 ml samples. The purified immunoprecipitated DNA was dissolved in 60 μl of 10 mM Tris-HCl (pH 8.0) 1 mM EDTA. Primer pairs are reported in Additional file 5. The primer pairs to beused in the ChIP assays were derived from the PCR analysis of panels of gorilla, chimpanzee and human somatic cell hybrids (from M. Rocchi and [44]). For the *FRG1 *gene, we derived five primer pairs that spanned 1500 bp upstream of the ATG, and produced amplification products only with the DNA from human chromosome 4. Using one such primer pair, the same result was obtained on panels of somatic cell hybrids containing gorilla and chimpanzee chromosomes. On the basis of these results, it can be assumed that this *FRG1 *primer pair is chromosome 4-specific in each of the analysed species (see example in Additional file 8), and so it was used for the ChIP assay of the *FRG1 *promoter. The primer pairs for the *FRG2 *promoter and 13E11 marker (shown in Additional file 5) were selected using a comparable approach. For both PCR reactions, an amplification product was observed only on the ape hybrids retaining chromosome 4, whereas an additional amplification band was detected on chromosome 10 in the human hybrids. However, on the basis of the ChIP analysis of a somatic cell hybrid retaining only chromosome 10, both the *FRG2 *promoter and 13E11 marker are very poorly histone 4 acetylated, a finding that allowed us to conclude that the high level of histone 4 acetylation observed in the human cells essentially comes from chromosome 4.

### TSA treatment

The HDAC inhibitor Trichostatin A (TSA, Sigma) was dissolved in EtOH and added to the culture medium at a final concentration of 330 nM for 16 h. An equal volume of EtOH was added to the medium of the untreated control cells.

### RNA isolation and real-time RT-PCR analysis

Total RNA was isolated from GGO, PTR and HSA lymphoblastoid cells using the RNA Wiz™ reagent (Ambion) according to the manufacturer's protocol. The integrity of the RNA was confirmed by denaturing gel electrophoresis. Real time RT-PCR analysis was performed on the iQ5 Real-Time PCR Detection System (BIORAD) using the iScriptTM One-Step RT-PCR Kit with SYBR^® ^Green (Biorad). The primers used for amplification are shown in Additional file 5. The specificity of the amplifications was assessed by means of electrophoretic separation of the amplified products and melting curve analysis. The relative expression of the investigated genes was quantified after normalisation with beta 2 microglobulin mRNA (B2M) [45,46]. All of the RT-PCR analyses were performed in triplicate in three independent experiments. The analyses made using primer pairs on the *FRG1 *and *FRG2 *promoters in human, chimpanzee and gorilla somatic cell hybrids strongly indicate that duplicated human and primate *FRG1 *and *FRG2 *genes carry divergent or incomplete promoter regions (with the exception of the *FRG2 *promoter on chromosome 10). However, RT-PCR analysis of a somatic cell hybrid retaining only chromosome 10 showed that the *FRG2 *gene is very poorly transcribed. These results allowed us to conclude that the transcripts we observed in each species were genuinely from chromosome 4.

## Abbreviations

FSHD: facioscapulohumeral muscular dystrophy; LINE: long interspersed nucleotide element; *FRG1 *and *FRG2*: FSHD region genes 1 and 2; *ANT1*: adenine nucleotide translocator 1 gene; GGO: *Gorila gorilla*; PTR: *Pan troglodytes*; PPA: *Pan paniscus*; HSA: *Homo sapiens*; BAC: bacterial artificial chromosome; PCR: polymerase chain reaction; RT-PCR: reverse transcriptase-PCR; FISH: fluorescent in situ hybridisation; ARR: average relative radius; CT: chromosome territory; ChIP: chromatin immunoprecipitation; TSA: trichostatin A; 3D-RRD: three dimensional relative radius distribution; ADS: enhanced absolute 3D distances to surfaces; B2M: b2-microglobulin gene; HDAC: histone deacetylase.

## Authors' contributions

BB: conceived and designed the experiments, performed the genomic molecular experiments, ChiP and RT-PCR assays, analysed the data, and wrote the manuscript; MFC: carried out the metaphase FISH experiments; SM: conceived and analysed the 3D-FISH experiments, and wrote the manuscript; MN: performed the confocal microscopy, 3D imaging and quantitative analyses; OF: performed dual color FISH on stretched metaphases chromosomes; ER: performed the ChiP and RT-PCR assays; EB: conceived the chromatin experiments, and critically analysed the ChIP data; AM: participated in critically reviewing the data; PR: planned and analysed the FISH experiments on stretched metaphases chromosomes; MR: conceived the FISH experiments, and critically analysed the genomic evolution data; RM: conceived and designed the experiments, and critically analysed the data; EG: as project supervisor, conceived and designed the experiments, analysed the data, and wrote the manuscript. All authors have read and approved the final manuscript.

## Supplementary Material

Additional File 1 **Supplementary Figure 1**. UCSC Human blat server [21] analysis of the location of gorilla BAC ends on chromosome 15q26.3, and partial overlapping on the same chromosome between the LINE block and subtelomeric block 3 [20]. **A) **Gorilla BAC ends (11C6-Sp6/T7, 18C5-Sp6/T7, 23B19-T7, 39M12-T7, 41H7-Sp6/T7) (GGO BAC ends, upper black rectangles) identify a LINE block of 35 kb on chromosome 15q26.3 (light grey bar). Similar repetitive blocks are also mapped on the 1p, 8p, and 19p subtelomeres. The repeat elements recognised by the Repeat Masker program are represented by rectangles in different shades of grey. **B) **Partial overlapping on chromosome 15q26.3 between the LINE block identified in A) and subtelomeric block 3 [20]. As in A), the repeat elements recognised by the Repeat Masker program are represented by rectangles in different shades of grey. The red rectangle (probe) identifies the location of the non-repetitive DNA sequence used as a probe for the screening of the gorilla genomic library. Click here for file

Additional File 2 **Supplementary Table 1**. Similarity of gorilla BAC ends with the human genome reference sequence. The Sp6 and T7 BAC ends of six gorilla clones were analysed for their similarity with the human genome reference sequence [21]; the table shows the most similar human chromosome regions. Click here for file

Additional File 3 **Supplementary Table 2**. Composition of the repeat sequences contained in the LINE block. Raw data obtained from Repeat Masker analysis of the LINE block repeat composition using Repeat Masker software [34]. Click here for file

Additional File 4 **Supplementary Figure 2**. Southern blot analysis of six D4Z4- and LINE-positive gorilla BAC clones. **A**) Agarose gel electrophoresis of EcoRI-digested DNA from six gorilla BACs (CH255-41H7, CH255-39N14, CH255-39M12, CH255-23B19, CH255-18C5 and CH255-11C6) positive for both D4Z4 and LINE sequences. Ethidium bromide staining (left), and Southern blot hybridisation with a LSau probe (right). **B**) Agarose gel electrophoresis of KpnI-digested DNA from BAC CH255-39M12 positive for both D4Z4 and LINE sequences. Ethidium bromide staining (OD left), and Southern blot hybridization (right) with LSau and beta satellite probes. M = molecular weight marker; bp = base pair. Click here for file

Additional File 5 **Supplementary Table 3**. Primer pairs used for sequencing, PCR, ChIP and RT-PCR. All the primer pairs used for the sequencing and PCR-based analyses are listed in the table, with their identification, 5'-3' sequence, and application. Click here for file

Additional File 6 **Supplementary Table 4**. Orthologous PCR on D4Z4- and LINE-positive gorilla BAC clones. Results of PCR amplification on the six gorilla BAC clones by various primer pairs identifying the FRG1 and FRG2 promoters and the 13E11 marker on 4qter, and 35 kb of the LINE block at the 15q subtelomere (primer pairs from 41H7-t7 to 23B19-T7). The nucleotide sequences of the used primer pairs are shown in Additional file 5. Click here for file

Additional File 7 **Supplementary Table 5**. Comparative FISH analyses of D4Z4- and LINE-positive gorilla genomic clones, and their sub-sequences. The table summarises the results of FISH on gorilla, chimpanzee and human metaphase chromosome spreads using the following probes: gorilla BACs (23B19-39N14-39M12-18C5), D4Z4 unit (subcloned form BAC 39M12), LINE block of 10 kb (amplified by PCR from BAC 39M12), and Inter-Alu PCR (from BACs 39M12 and 18C5). Click here for file

Additional File 8 **Supplementary Figure 3**. Amplification of *FRG1 *primer pair 1 (*FRG1*pr1) on a panel of human-rodent somatic cell hybrids, each containing a single human chromosome. Example of PCR screening performed to test the chromosome specificity of the regions analysed by the ChiP assay; the panel of human, chimpanzee and gorilla somatic cell hybrids were provided by M. Rocchi and [44], and used as the DNA template for PCR analyses of the *FRG1 *promoter, the *FRG2 *promoter, and the 13E11 marker. The *FRG1*, *FRG2 *and 13E11 PCR primer pairs were derived from human chromosome 4 databank sequences [47], and are shown in Additional file 5. Roman numbers identify the human chromosomes in each somatic cell hybrid. C+ = positive control; C- = negative control. Click here for file
