# Supplementary figures and images for: Evolutionary genomic remodelling of the human 4q subtelomere (4q35.2)
Source: BMC Evol Biol. 2007 Mar 14;7:39. doi: 10.1186/1471-2148-7-39 (PMC1852401; doi:10.1186/1471-2148-7-39)

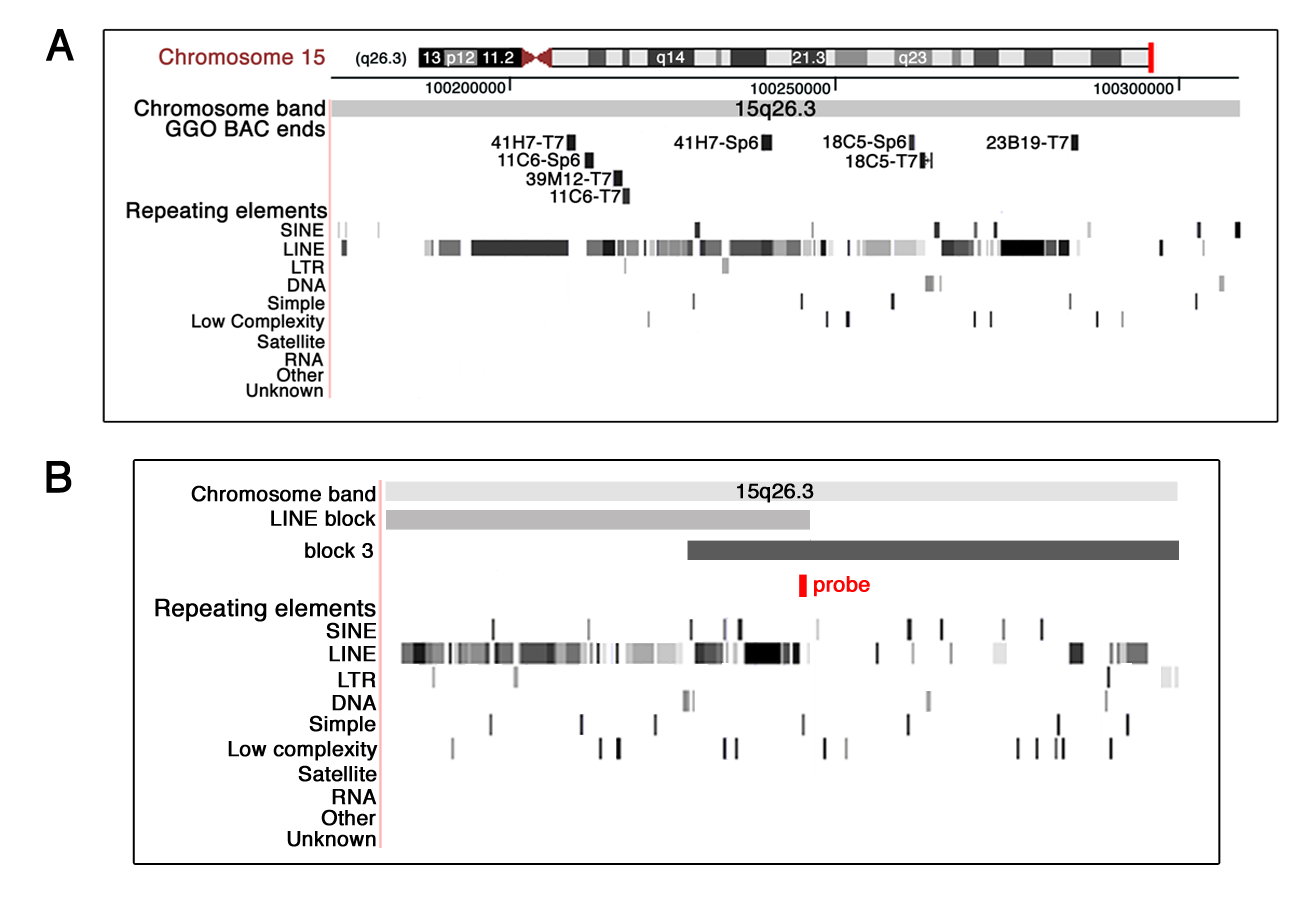

Supplement: Additional File 1 — Supplementary Figure 1. UCSC Human blat server [21] analysis of the location of gorilla BAC ends on chromosome 15q26.3, and partial overlapping on the same chromosome between the LINE block and subtelomeric block 3 [20]. A) Gorilla BAC ends (11C6-Sp6/T7, 18C5-Sp6/T7, 23B19-T7, 39M12-T7, 41H7-Sp6/T7) (GGO BAC ends, upper black rectangles) identify a LINE block of 35 kb on chromosome 15q26.3 (light grey bar). Similar repetitive blocks are also mapped on the 1p, 8p, and 19p subtelomeres. The repeat elements recognised by the Repeat Masker program are represented by rectangles in different shades of grey. B) Partial overlapping on chromosome 15q26.3 between the LINE block identified in A) and subtelomeric block 3 [20]. As in A), the repeat elements recognised by the Repeat Masker program are represented by rectangles in different shades of grey. The red rectangle (probe) identifies the location of the non-repetitive DNA sequence used as a probe for the screening of the gorilla genomic library. [file 1471-2148-7-39-S1.tiff]

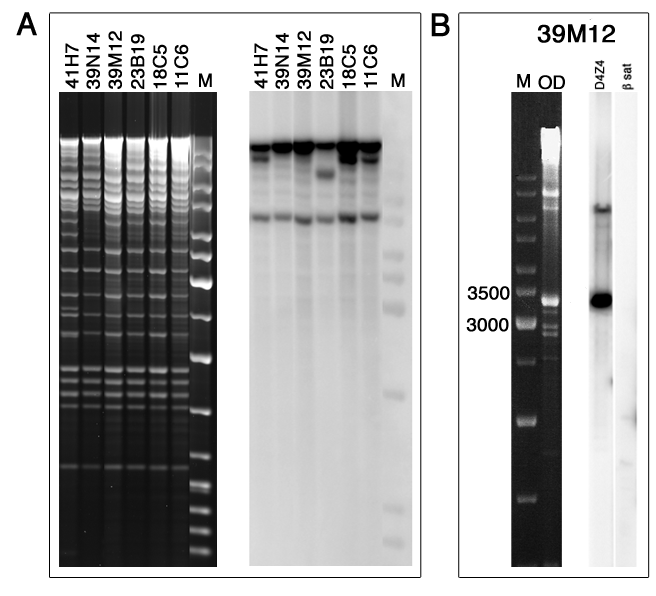

Supplement: Additional File 4 — Supplementary Figure 2. Southern blot analysis of six D4Z4- and LINE-positive gorilla BAC clones. A) Agarose gel electrophoresis of EcoRI-digested DNA from six gorilla BACs (CH255-41H7, CH255-39N14, CH255-39M12, CH255-23B19, CH255-18C5 and CH255-11C6) positive for both D4Z4 and LINE sequences. Ethidium bromide staining (left), and Southern blot hybridisation with a LSau probe (right). B) Agarose gel electrophoresis of KpnI-digested DNA from BAC CH255-39M12 positive for both D4Z4 and LINE sequences. Ethidium bromide staining (OD left), and Southern blot hybridization (right) with LSau and beta satellite probes. M = molecular weight marker; bp = base pair. [file 1471-2148-7-39-S4.tiff]
