# Supplementary material for: Evolutionary genomic remodelling of the human 4q subtelomere (4q35.2)
Source: BMC Evol Biol. 2007 Mar 14;7:39. doi: 10.1186/1471-2148-7-39 (PMC1852401; doi:10.1186/1471-2148-7-39)
Supplement: Additional File 2 — Supplementary Table 1. Similarity of gorilla BAC ends with the human genome reference sequence. The Sp6 and T7 BAC ends of six gorilla clones were analysed for their similarity with the human genome reference sequence [21]; the table shows the most similar human chromosome regions. [file 1471-2148-7-39-S2.doc]

| **BAC clone** | **Sp6** | **T7** |
| --- | --- | --- |
| **11C6** | 15q26.3** | 15q26.3** |
| **18C5** | 15q26.3** | 15q26.3** |
| **23B19** | 4q35.2* | 15q26.3** |
| **39M12** | 4q35.2* | 15q26.3** |
| **39N14** | No similarity | 4q35.2* |
| **41H7** | 15q26.3** | 15q26.3** |

Supplementary Table 1 - Similarity of gorilla BAC ends with the human genome data bank

* at 4q35.2, clustered in approx. 15kb, distal to the *FRG2* gene (clones AF146191-U85056)

** clustered in approx. 35kb of 15q and 19p subtelomeres (clones AC107977-AC140725 for chromosome 15, and clones AC008977-AC016626 for chromosome 19)
