# Supplementary material for: Evolutionary genomic remodelling of the human 4q subtelomere (4q35.2)
Source: BMC Evol Biol. 2007 Mar 14;7:39. doi: 10.1186/1471-2148-7-39 (PMC1852401; doi:10.1186/1471-2148-7-39)
Supplement: Additional File 3 — Supplementary Table 2. Composition of the repeat sequences contained in the LINE block. Raw data obtained from Repeat Masker analysis of the LINE block repeat composition using Repeat Masker software [34]. [file 1471-2148-7-39-S3.doc]

Supplementary Table 2 - Row data from Repeat Masker analysis of LINE block repeat composition using the software http://repeatmasker.org SW perc perc perc position in query matching repeat position in repeat

score div. del. ins. begin end (left) repeat class/family begin end (left) ID

5095 16.9 3.1 3.5 1 1073 (32037) C L1M3 LINE/L1 (1415) 4725 3657 1

1443 4.5 0.0 0.0 1097 1275 (31835) C L1PA4 LINE/L1 (0) 6155 5977 2

6111 3.2 0.0 0.0 1276 1997 (31113) + L1PA4 LINE/L1 5254 5975 (179) 2

2271 19.3 1.0 0.2 2015 2507 (30603) C L1M3 LINE/L1 (2494) 3646 3150 1

462 23.1 9.9 0.0 2509 2629 (30481) C ERVL LTR/ERVL (3935) 1822 1690 3

3490 18.7 6.9 0.7 2637 3464 (29646) C L1M2 LINE/L1 (2326) 3817 2939 4

786 4.1 0.0 0.0 3913 4010 (29100) C L1PA5 LINE/L1 (2) 6152 6055 5

292 23.9 3.2 2.1 4097 4190 (28920) + CT-rich Low_complexity 2 96 (0) 6

564 26.2 8.6 6.5 4198 4629 (28481) + L1MEd LINE/L1 708 1148 (4971) 7

1305 13.1 3.3 0.5 4655 4869 (28241) C L1PA10 LINE/L1 (2) 6166 5946 8

329 18.2 7.8 0.0 4870 4946 (28164) C L1PA8 LINE/L1 (144) 6028 5946 9 *

333 22.7 6.1 1.0 4926 5023 (28087) C L1PA8 LINE/L1 (124) 6048 5946 9 *

356 18.8 7.1 2.0 5003 5100 (28010) C L1PA8 LINE/L1 (124) 6048 5946 9

354 21.6 6.1 1.0 5080 5177 (27933) C L1PA8 LINE/L1 (124) 6048 5946 9 *

326 21.9 7.2 1.0 5157 5253 (27857) C L1PA8 LINE/L1 (124) 6048 5946 9 *

347 19.8 7.1 2.0 5233 5330 (27780) C L1PA8 LINE/L1 (124) 6048 5946 9 *

364 20.6 6.1 1.0 5310 5407 (27703) C L1PA8 LINE/L1 (124) 6048 5946 9

333 22.7 6.1 1.0 5387 5484 (27626) C L1PA8 LINE/L1 (124) 6048 5946 9 *

323 23.7 6.1 1.0 5464 5561 (27549) C L1PA8 LINE/L1 (124) 6048 5946 9 *

334 19.8 7.1 2.0 5541 5638 (27472) C L1PA8 LINE/L1 (124) 6048 5946 9 *

342 21.6 6.1 1.0 5618 5715 (27395) C L1PA8 LINE/L1 (124) 6048 5946 9

342 21.6 6.1 1.0 5695 5792 (27318) C L1PA8 LINE/L1 (124) 6048 5946 9

325 20.8 7.1 2.0 5772 5869 (27241) C L1PA8 LINE/L1 (124) 6048 5946 9 *

356 18.8 7.1 2.0 5849 5946 (27164) C L1PA8 LINE/L1 (124) 6048 5946 9

348 22.7 6.1 1.0 5926 6023 (27087) C L1PA8 LINE/L1 (124) 6048 5946 9 *

313 22.9 6.2 1.0 6003 6099 (27011) C L1PA8 LINE/L1 (124) 6048 5947 9 *

316 22.7 6.1 1.0 6080 6177 (26933) C L1PA8 LINE/L1 (124) 6048 5946 9 *

385 19.6 6.1 1.0 6157 6254 (26856) C L1PA8 LINE/L1 (124) 6048 5946 9

346 21.9 6.2 1.0 6234 6330 (26780) C L1PA8 LINE/L1 (124) 6048 5947 9 *

294 23.7 6.1 1.0 6311 6408 (26702) C L1PA8 LINE/L1 (124) 6048 5946 9 *

357 19.8 7.2 1.0 6388 6484 (26626) C L1PA8 LINE/L1 (124) 6048 5946 9 *

374 20.6 6.1 1.0 6464 6561 (26549) C L1PA8 LINE/L1 (124) 6048 5946 9

356 18.8 7.1 2.0 6541 6638 (26472) C L1PA8 LINE/L1 (124) 6048 5946 9 *

343 21.6 6.1 1.0 6618 6715 (26395) C L1PA8 LINE/L1 (124) 6048 5946 9 *

2320 11.2 0.5 1.2 6676 7067 (26043) C L1PA8A LINE/L1 (77) 6077 5675 9

533 24.3 1.1 4.2 6917 7105 (26005) C L1ME3A LINE/L1 (348) 5825 5643 9 *

830 14.3 0.7 0.0 7106 7252 (25858) + (TA)n Simple_repeat 1 148 (0) 10

2114 12.8 0.0 0.0 7253 7542 (25568) C AluSq SINE/Alu (23) 290 1 11

2717 10.2 3.2 0.0 7545 7954 (25156) C L1P4 LINE/L1 (478) 5690 5268 12 *

6224 18.7 0.3 0.8 7948 8988 (24122) C L1PA15-16 LINE/L1 (5816) 1037 2 13

1788 21.5 7.6 1.5 9029 9504 (23606) C LTR22C LTR/ERVK (4) 505 1 14

234 26.8 5.0 0.7 9522 9660 (23450) + L1ME3B LINE/L1 3224 3368 (2778) 15 *

7098 15.5 2.9 0.7 9659 11640 (21470) + L1MA8 LINE/L1 4080 6111 (180) 16

4906 10.3 1.8 0.1 11641 12376 (20734) C L1MA2 LINE/L1 (1) 6303 5556 17

5091 15.9 4.2 1.4 12377 12500 (20610) + L1MA8 LINE/L1 6112 6238 (53) 16

1614 25.4 7.3 3.2 12535 13272 (19838) + L1ME3B LINE/L1 3352 4119 (2027) 15

5050 17.2 2.7 2.9 13324 14336 (18774) + L1P4 LINE/L1 3687 4697 (1449) 18

186 4.3 0.0 0.0 14337 14359 (18751) + (CAAAA)n Simple_repeat 2 24 (0) 19

1194 30.7 8.2 2.6 14395 14976 (18134) + L1ME3B LINE/L1 5508 6122 (118) 15

694 19.9 9.3 0.6 15003 15164 (17946) C AluSp SINE/Alu (15) 298 123 20

229 26.0 0.0 1.3 15170 15247 (17863) + L1ME3B LINE/L1 6128 6204 (36) 15

343 37.1 6.2 2.2 15416 15641 (17469) + L2 LINE/L2 2579 2813 (606) 21

2192 4.1 0.8 0.0 15645 15912 (17198) + L1PA5 LINE/L1 5881 6150 (4) 22

25 3.1 0.0 0.0 15973 16004 (17106) + AT_rich Low_complexity 1 32 (0) 23

609 31.9 15.6 0.9 16065 16384 (16726) + L2c LINE/L2 2980 3346 (41) 21

24 6.5 0.0 1.6 17329 17357 (15753) + AT_rich Low_complexity 1 30 (0) 24

389 4.3 0.0 0.0 17358 17404 (15706) + L1PA6 LINE/L1 6100 6146 (8) 25

24 6.5 0.0 1.6 17405 17437 (15673) + AT_rich Low_complexity 1 32 (0) 26

375 28.5 8.4 3.4 18008 18185 (14925) C L1ME3B LINE/L1 (0) 6240 6054 27

455 29.9 6.4 2.3 18308 18571 (14539) C L1MC5 LINE/L1 (436) 7525 7251 28

4153 20.5 8.2 3.8 18638 20264 (12846) C L1M4c LINE/L1 (2924) 3720 2017 29

1150 6.2 0.5 3.2 20265 20449 (12661) + (TTTC)n Simple_repeat 1 180 (0) 30

3473 23.7 11.2 7.3 20450 21982 (11128) C L1M4c LINE/L1 (4630) 2016 471 29

523 29.1 8.9 4.7 21998 22567 (10543) C L1M5 LINE/L1 (2584) 3562 2969 31

2325 17.9 6.7 0.7 22570 23029 (10081) C Tigger5 DNA/MER2_type (0) 2406 1919 32

1033 23.4 1.8 6.5 23030 23164 (9946) C Tigger5 DNA/MER2_type (2188) 218 89 32

2243 8.3 0.3 0.7 23165 23456 (9654) C AluSc SINE/Alu (14) 295 5 33

1033 23.4 1.8 6.5 23457 23549 (9561) C Tigger5 DNA/MER2_type (2318) 88 1 32

5525 8.1 0.9 1.0 23627 24426 (8684) C L1PB LINE/L1 (838) 5308 4510 34 *

5763 15.2 3.8 0.2 24406 25365 (7745) C L1PREC2 LINE/L1 (5704) 2451 1458 35

1985 19.4 1.2 0.9 25366 25797 (7313) C L1M2 LINE/L1 (3549) 2594 2162 36

22 0.0 0.0 0.0 25802 25823 (7287) + AT_rich Low_complexity 1 22 (0) 37

778 19.7 0.0 0.0 25829 25970 (7140) C FLAM_C SINE/Alu (1) 142 1 38

259 8.2 1.9 5.8 25973 26024 (7086) C L1M2 LINE/L1 (3971) 2878 2829 39 *

425 24.5 6.1 4.1 26021 26314 (6796) C L1MC5 LINE/L1 (315) 7646 7347 40

28 2.9 0.0 0.0 26817 26851 (6259) + AT_rich Low_complexity 1 35 (0) 41

904 24.7 12.8 2.8 26868 27055 (6055) C L1MC5 LINE/L1 (1940) 5942 5735 40

2190 13.1 0.0 0.0 27056 27360 (5750) C AluSx SINE/Alu (7) 305 1 42

904 24.7 12.8 2.8 27361 27563 (5547) C L1MC5 LINE/L1 (2148) 5734 5512 40 *

15293 2.3 0.0 0.0 27559 29358 (3752) C L1PA2 LINE/L1 (1036) 5110 3311 43

7804 2.0 0.0 0.0 29361 30411 (2699) + L1PA2 LINE/L1 5105 6155 (0) 43

438 23.5 3.2 4.5 30416 30571 (2539) C L1MC5 LINE/L1 (2357) 5525 5372 40

3738 18.0 0.6 0.1 30581 31249 (1861) + L1PA16 LINE/L1 5491 6162 (4) 44

2959 5.7 1.1 0.0 31361 31732 (1378) C L1PA7 LINE/L1 (364) 5790 5415 45

2669 6.5 1.5 0.0 31733 32073 (1037) + L1PA7 LINE/L1 5805 6150 (4) 45

474 17.8 0.0 0.0 32095 32184 (926) + (TATG)n Simple_repeat 3 92 (0) 46

398 34.8 5.4 4.7 32626 32902 (208) C L1MC5 LINE/L1 (1058) 5088 4810 40
