# Supplementary material for: Evolutionary genomic remodelling of the human 4q subtelomere (4q35.2)
Source: BMC Evol Biol. 2007 Mar 14;7:39. doi: 10.1186/1471-2148-7-39 (PMC1852401; doi:10.1186/1471-2148-7-39)
Supplement: Additional File 6 — Supplementary Table 4. Orthologous PCR on D4Z4- and LINE-positive gorilla BAC clones. Results of PCR amplification on the six gorilla BAC clones by various primer pairs identifying the FRG1 and FRG2 promoters and the 13E11 marker on 4qter, and 35 kb of the LINE block at the 15q subtelomere (primer pairs from 41H7-t7 to 23B19-T7). The nucleotide sequences of the used primer pairs are shown in Additional file 5. [file 1471-2148-7-39-S6.doc]

Supplementary Table 4- Paralogous PCR on D4Z4- and LINE-positive gorilla BAC clones by primer pairs defining DNA regions proximal to the human 4q35.2 D4Z4 array (FRG1, FRG2 and 13E11) and 35 kb of the LINE block at the 15q subtelomere (from 41H7 T7 to 23B19 T7).

| GGO Clones | FRG1 | FRG2 | 13E11 | 41H7  T7 | 11C6  Sp6 | 39M12  T7 | 11C6  T7 | 41H7  Sp6 | 18C5  Sp6 | 18C5  T7 | 23B19  T7 |
| --- | --- | --- | --- | --- | --- | --- | --- | --- | --- | --- | --- |
| 11C6 | - | + | + | + | + | + | + | + | - | + | + |
| 18C5 | - | - | + | + | + | + | + | + | + | + | + |
| 23B9 | - | + | + | + | + | + | + | + | + | + | + |
| 39M12 | - | - | + | + | + | + | + | + | + | + | + |
| 39N14 | - | + | + | + | + | + | + | + | + | + | + |
| 41H7 | - | + | + | + | + | + | + | + | + | + | + |

Primer pairs for PCR amplification are listed in Additional File 6: Supplementary Table 3.
