# Supplementary material for: Evolutionary genomic remodelling of the human 4q subtelomere (4q35.2)
Source: BMC Evol Biol. 2007 Mar 14;7:39. doi: 10.1186/1471-2148-7-39 (PMC1852401; doi:10.1186/1471-2148-7-39)
Supplement: Additional File 7 — Supplementary Table 5. Comparative FISH analyses of D4Z4- and LINE-positive gorilla genomic clones, and their sub-sequences. The table summarises the results of FISH on gorilla, chimpanzee and human metaphase chromosome spreads using the following probes: gorilla BACs (23B19-39N14-39M12-18C5), D4Z4 unit (subcloned form BAC 39M12), LINE block of 10 kb (amplified by PCR from BAC 39M12), and Inter-Alu PCR (from BACs 39M12 and 18C5). [file 1471-2148-7-39-S7.doc]

| Clone | **GGO** | **PTR** | HSA |
| --- | --- | --- | --- |
| 23B19 | 3p-4qter | 3p-4qter | Many loci, including 4qter and the p arm of acrocentrics |
| 39N14 | 3p-4qter | 3p-4qter | Many loci, including 4qter and the p arm of acrocentrics |
| 39M12 | 3p-4qter | 3p-4qter | Many loci, including 4qter and the p arm of acrocentrics |
| 18C5 | 3p-4qter-22p | 3p/pter-4qter | Many loci, including 4qter and the p arm of acrocentrics |
| 39M12-D4Z4 | 3p-4p/qter | 3p-4qter-13p-14p-15p-21p-22p | 1qcen-4qter-10qter-13p-14p-15p-21p-22p |
| 39M12-LINE | 4qter | 4qter | 1pter-8pter-15qter-19pter |
| 39M12-I.A.* | 4qter | 4qter | 4qter-10qter |
| 18C5-I.A.* | 4qter | 4qter | 4qter-10qter |

Supplementary Table 5 - Comparative FISH with D4Z4- and LINE-positive gorilla genomic clones

GGO (gorilla), PTR (chimpanzee) and HSA (humans)

* I.A. = inter-Alu sequences
